# 

**Published:** 1881-06

**Authors:** 


					﻿WITH SUPPLEMENT
Whole Number,
CCCCXXXVII.
June, 1881.
Number 6,
Vol. XLII.
THE
CHICAGO
MEDIC AL
T ournal and Examiner
EDITORS:
SEN. S. DAVIS, M.D., LL.D. JAS. NEVINS HYDE, A.M., M.D. 15T5
DANIEL R. BROWER, M.D.
CHICAGO:
PUBLISHED BY THE CHICAGO MEDICAL PRESS ASSOCIATION,
188 Clark Street.
^*Read the Notice of this Journal among Advertisements*
				

